# Dietary Intake and Energy Expenditure in Breast Cancer Survivors: A Review

**DOI:** 10.3390/nu13103394

**Published:** 2021-09-27

**Authors:** Sarah A. Purcell, Ryan J. Marker, Marc-Andre Cornier, Edward L. Melanson

**Affiliations:** 1 Division of Endocrinology, Metabolism, and Diabetes, Department of Medicine, Anschutz Medical Campus, University of Colorado, Aurora, CO 80045, USA; marc.cornier@cuanschutz.edu (M.-A.C.); ed.melanson@cuanschutz.edu (E.L.M.); 2 Anschutz Health and Wellness Center, Anschutz Medical Campus, University of Colorado, Aurora, CO 80045, USA; ryan.marker@cuanschutz.edu; 3 Department of Physical Medicine and Rehabilitation, Anschutz Medical Campus, University of Colorado, Aurora, CO 80045, USA; 4 Rocky Mountain Regional VA Medical Center, Aurora, CO 80045, USA; 5 Division of Geriatric Medicine, Department of Medicine, Anschutz Medical Campus, University of Colorado, Aurora, CO 80045, USA

**Keywords:** metabolism, obesity, nutrition, exercise, oncology

## Abstract

Many breast cancer survivors (BCS) gain fat mass and lose fat-free mass during treatment (chemotherapy, radiation, surgery) and estrogen suppression therapy, which increases the risk of developing comorbidities. Whether these body composition alterations are a result of changes in dietary intake, energy expenditure, or both is unclear. Thus, we reviewed studies that have measured components of energy balance in BCS who have completed treatment. Longitudinal studies suggest that BCS reduce self-reported energy intake and increase fruit and vegetable consumption. Although some evidence suggests that resting metabolic rate is higher in BCS than in age-matched controls, no study has measured total daily energy expenditure (TDEE) in this population. Whether physical activity levels are altered in BCS is unclear, but evidence suggests that light-intensity physical activity is lower in BCS compared to age-matched controls. We also discuss the mechanisms through which estrogen suppression may impact energy balance and develop a theoretical framework of dietary intake and TDEE interactions in BCS. Preclinical and human experimental studies indicate that estrogen suppression likely elicits increased energy intake and decreased TDEE, although this has not been systematically investigated in BCS specifically. Estrogen suppression may modulate energy balance via alterations in appetite, fat-free mass, resting metabolic rate, and physical activity. There are several potential areas for future mechanistic energetic research in BCS (e.g., characterizing predictors of intervention response, appetite, dynamic changes in energy balance, and differences in cancer sub-types) that would ultimately support the development of more targeted and personalized behavioral interventions.

## 1. Introduction

Breast cancer prevention, screening practices, and effective treatment modalities confer favorable long-term survival in breast cancer survivors (BCS). In fact, 62% of cases are diagnosed at a localized stage (no spread to locations outside the breast), for which the 5-year survival is 99% [1]. Despite high success rates of breast cancer treatment, many cancer survivors have increased risk of developing comorbidities such as cardiovascular disease, diabetes, and chronic pain compared to age-matched women without previous cancer [2,3]. Unfavorable body composition profiles (i.e., reduced fat-free mass [FFM], increased fat mass [FM]) may contribute to the development of comorbidities and poorer survival. In fact, over one-third of women with nonmetastatic breast cancer may have low FFM at diagnosis, which is associated with higher overall mortality, especially when this occurs with high FM, or ‘sarcopenic obesity’ [4]. Changes in body composition may also worsen during chemotherapy, radiation, and surgery or long-term estrogen suppression therapy [5,6,7]. The effects on body composition may persist years after completion of treatment and negatively impacts long-term prognosis and risk of developing comorbidities [4,8,9,10,11]. In fact, up to 28% of metastatic [12] and 6% of non-metastatic [13] BCS with obesity have low FFM. Characterizing the mechanisms that contribute to changes in body composition in BCS would guide intervention strategies to improve overall health of this population.

Fundamentally, alterations in body composition are indicative of long-term energy balance induced by changes in dietary intake (e.g., energy intake [EI], macronutrient intake), and/or total daily energy expenditure (TDEE; primarily resting metabolic rate [RMR] and physical activity energy expenditure). However, mechanisms contributing to changes in energy balance in BCS are largely uncharacterized. Premenopausal BCS often undergo long-term therapeutic estrogen suppression; this treatment modality may increase the likelihood of elevated EI and/or decreased TDEE. However, our understanding of estrogen in energy balance arises from experimental estrogen suppression studies in women without previous breast cancer. Therefore, characterizing energy balance components is particularly relevant in this population as this could support the generation of targeted interventions that prevent adverse alterations in body composition.

Understanding how dietary intake and energy expenditure changes independently of behavioral interventions and whether components of energy balance in BCS differ compared to healthy controls would help support the generation of personalized behavioral weight management programs and dietary and physical activity guidelines. Therefore, the objective of this review is to summarize studies that have characterized changes in dietary intake and energy expenditure in BCS after completion of treatment (i.e., chemotherapy, radiation, and surgery). Because estrogen suppression therapy may have independent effects on dietary intake and energy expenditure, we also review evidence from studies that have performed experimental suppression of ovarian function to provide insight into potential mechanisms contributing to energy imbalance in BCS.

## 2. Materials and Methods

To enhance the rigor of this review, a search was conducted in PubMed and Web of Science to identify articles that characterized dietary intake, energy expenditure, and/or physical activity in BCS from inception until 26 May 2021. Search terms included those related to “breast cancer” and “diet”, “energy expenditure”, or “physical activity” (Table 1). Only peer-reviewed articles published in English were included. Bibliographies of each included article were searched for other potentially relevant literature. Studies were included if changes in dietary intake, energy expenditure, or physical activity were measured in BCS at diagnosis or at completion of treatment (chemotherapy, radiation, and surgery) with any duration of follow-up. In addition, studies that included a comparison between BCS after treatment and control subjects were reviewed. Studies that measured dietary intake or energy expenditure parameters only at diagnosis and during treatment (or assumed treatment if follow-up was <6 months from diagnosis) were excluded. While this review would ideally discuss the independent effects of treatment and long-term estrogen suppression therapy, there is a lack of data disentangling the impact of these modalities on energy balance. We did not exclude literature based on breast cancer sub-types or stage due to the limited number of observational studies identified. This review also focuses primarily on the effects of estrogen-receptor positive breast cancer, because this is the most commonly diagnosed form of breast cancer, there is a smaller body of literature specifically in estrogen receptor-negative BCS, and estrogen suppression therapy may have lasting effects on energy balance. In addition, only studies of female BCS were uncovered in our search and included in this review.

## 3. Results

### 3.1. Dietary Intake in Breast Cancer Survivors

Dietary intake is half of the energy balance equation, and therefore essential to characterize across populations and settings. Describing dietary intake is particularly important after cancer treatment as prudent/healthy dietary patterns (high intake of fruits, vegetables, whole grains, poultry, and low-fat dairy) are associated with lower mortality and Western-style dietary patterns (high intake of red and processed meats, refined grains, sweets and desserts, and high-fat dairy products) are associated with higher mortality in BCS [14]. As such, current recommendations for BCS encourage a predominantly plant-based diet that is low in fat, rich in fruits, vegetables, and whole grains, and supports a healthy body weight [15].

To help BCS meet these goals, it is necessary to characterize dietary intake longitudinally as well as in comparison to women without previous cancer. We identified six studies that measured dietary intake across the survivorship trajectory [16,17,18,19,20,21], with one also comparing dietary intake to healthy control subjects [17], Table 2. Sample sizes ranged from 116 to 2865 women and included BCS with both estrogen-receptor positive and negative tumors and all stages of disease (although there was a preponderance of non-metastatic cancer). All studies measured dietary intake using food frequency questionnaires (FFQs).

As summarized in Table 2, dietary changes were presented as nutrients and/or food groups. Five studies reported decreased EI [16,17,18,20,21] in BCS with concomitant decreased absolute fat intake (g/day) [16,17,18,20] in four studies and decreased relative fat intake (as a percent of total EI) [17,18,21] in three studies. Four studies reported decreased absolute protein intake in BCS [16,18,20,21]; however, this may be a result of decreased EI, as two studies reported increased [17] or no change [18] in protein when expressed as percent of total EI. Changes in carbohydrate intake were inconsistent, with studies reporting increased [16,17,18], decreased [17,20,21], or no change [18] in carbohydrate intake expressed in absolute or relative terms.

Five studies measured dietary intake as changes in food groups. There was a general increase in the amount of fruit and vegetables consumed [16,17,19,20] and decrease in red meat consumption [16,20] after treatment. No other discernable and consistent patterns in food-based dietary intake were observed, which may be partially due to the different methods of grouping foods together. The study that also assessed dietary intake in BCS compared to controls [17] found only slightly lower fiber (17.4 vs. 18.7 g/day, *p* = 0.04) and percentage of EI from alcohol (1.1 vs. 2.5%, *p* = 0.005) intake in BCS [17].

These results reported few changes toward unhealthy dietary intake patterns and suggest that there is generally a shift towards healthier dietary intake in the late survivorship phase. This aligns with other investigations that have utilized general retrospective questionnaires about changes in dietary intake following breast cancer diagnosis. For example, a study of 123 Finnish women reported that 31.9% had made changes to their diet after diagnosis, primarily through reduced animal fat, sugar, and red meat and increased fruit and vegetables [22]. Similarly, a sample of 3084 American BCS reported increasing intake of fruits (58% of the sample), vegetables (60%), and whole grains (39%) and decreasing intake of red meat (61%), cheese (53%), and fast foods (49%) after diagnosis [23]. Comparable positive dietary changes were noted in a sample of 28 Canadian BCS in the first year after chemotherapy, although many women were still below the recommendations for fruit, vegetables, milk and milk alternatives, calcium, and vitamin D [24].

A major limitation of these studies is reliance on FFQs. FFQs capture long-term intake (usually 12 months) of food groups and may therefore provide insight into general alterations of dietary patterns. Nevertheless, FFQs have poor accuracy for determining EI, macronutrients, and micronutrients [25,26]. Dietary recall and record methods are dependent on participant memory and ability to accurately assess dietary intake. In fact, BCS may underreport EI (especially from less “socially desirable” foods) in a similar manner to adults without previous cancer. For example, among 1137 BCS in the Women’s Healthy Eating and Living Survey, 25.6% were classified as low-energy reporters according to the Goldberg cut points [27] from estimated TDEE and 24-h recalls [28]; BCS with obesity or a history of weight gain or weight fluctuations were more likely to be low energy reporters. It is therefore likely that FFQ-based assessments do not reflect actual changes in energy and macronutrient intake. The data presented in this review precluded estimation of EI change via mathematical approaches using changes in body energy stores (e.g., energy intake-balance method [29,30]). It is also important to view changes in dietary intake in the context of overall dietary patterns because a single food item or nutrient is unlikely to yield substantial changes in energy balance over the long term. Furthermore, many of the reported changes were small in magnitude, and the ultimate impact on energy balance is unknown. Despite these limitations, there was a general trend in lower EI and improved dietary patterns across several studies, which suggests that at least some FM gain in BCS may be a result of changes in energy expenditure.

### 3.2. Energy Expenditure in Breast Cancer Survivors

Total daily energy expenditure is comprised of RMR, physical activity energy expenditure, and the thermic effect of food (TEF). RMR is the largest component of TDEE and is primarily determined by FFM. Physical activity energy expenditure consists of exercise and non-exercise physical activity. TEF is determined by EI and macronutrient composition and often assumed to be 10% of TDEE. Characterizing changes in the components of TDEE in BCS during estrogen suppression therapy would help inform EI and physical activity guidelines in this population. Therefore, this review discusses studies that measured RMR and physical activity in BCS (no published studies have reported changes in TEF in BCS after treatment).

Many BCS lose FFM [4,6,31], which would be expected to contribute to a reduction in RMR. During chemotherapy, RMR tends to decrease mid-treatment, but rebound to pre-treatment levels at the end of treatment [32,33]. Some evidence suggests that RMR changes in accordance with FFM alterations during treatment [34], although this has not been consistently observed [33]. To our knowledge, no study has longitudinally assessed RMR changes in BCS after completion of treatment. However, a recent cross-sectional study evaluated RMR in 17 BCS (body mass index [BMI]: 26.4 ± 5.1 kg/m^2^; age: 59 ± 9 years) who had completed treatment for stage I-III cancer 76 ± 18 months prior compared to 18 age-matched postmenopausal women (BMI: 25.3 ± 3.8 kg/m^2^; age 59 ± 6 years) [35]. There were no differences in body composition measured by dual X-ray absorptiometry, but there was a trend toward increased absolute RMR in BCS compared to controls (1381 ± 191 vs. 1270 ± 184 kcal/day, *p* = 0.099). When RMR was divided by lean soft tissue (FFM − bone), BCS had significantly higher values of adjusted RMR (36.1 ± 2.2 vs. 33.0 ± 4.3, *p* = 0.015) [35]. Although speculative, increased RMR may be due to lasting systemic inflammation [36,37,38] or insulin resistance [39]. It is also important to note that higher RMR does not necessarily confer increased TDEE; it is possible that increases in RMR could be offset by changes in other components of TDEE. Specifically, attributing changes in energy balance to altered RMR assumes that all other components of TDEE remain constant. As the body of physical activity literature presented in this article suggest [40,41,42,43], RMR alterations maybe negated by decreased physical activity. For context, a 110 kcal/day increase in RMR (as observed in the study described above [35]) is equivalent to 31 min of moderately-paced (2.8–3.2 mph or 4.5–5.2 kph) walking for a 70 kg person (3.5 metabolic equivalency of tasks) [44]. It is therefore conceivable that alterations in physical activity may nullify or augment changes in RMR.

Physical activity energy expenditure is the component of TDEE that can be modulated by behavior and is highly heterogeneous among populations and individuals. Physical activity may change after a diagnosis and treatment for breast cancer. On one hand, BCS have reported that physical activity can enhance feelings of empowerment and facilitate weight management [45]; conversely, many BCS have lasting fatigue [46], which is a major barrier to exercise engagement [47]. Therefore, assessing physical activity in BCS is important to facilitate personalized and effective behavioral interventions.

Because self-reported physical activity differs from objectively measured physical activity among BCS [48] this review only includes studies that utilized objective measures of physical activity (i.e., accelerometers) rather than self-reported data, Table 3. Six studies were included [40,41,42,43,49,50]; of these, two assessed physical activity longitudinally [40,42], four compared physical activity to controls without previous cancer [41,43,49,50], and one study reported physical activity longitudinally and in comparison to controls [40]. Breast cancer survivors had decreased light activity over time [40] and lower light activity compared to controls [40,41]. Findings of other components of physical activity and sedentary behavior were not consistent. For example, sedentary time increased longitudinally or was higher compared to controls in two studies [40,41], but remained unchanged [42] or lower than controls [50] in other investigations. Moderate-to-vigorous physical activity generally decreased [42] or was lower than controls [41,43], although this was not a consistent finding [49]. Collectively, objectively-measured physical activity appears to decrease with concomitant increases in sedentary time, although this is not universal across studies.

An evident gap in the literature is the lack of objectively measured TDEE among BCS. In fact, only five studies have assessed TDEE using objective measures in patients with cancer [51,52,53,54,55], although there are at least two other ongoing studies measuring TDEE using doubly labeled water or whole-room calorimetry in cancer survivors, one of which is being conducted in BCS (clinicaltrials.gov (accessed on 6 September 2021) identifiers: NCT0092961 and NCT02788955). The reliance on RMR and lack of existing TDEE data using doubly labeled water limits our ability to formulate a comprehensive understanding of energy balance and how these may be altered as a result of cancer, treatment modalities, or long-term estrogen suppression therapy. It is therefore unclear if BCS have different TDEE (and therefore EI requirements) compared with women without previous cancer or if short- or long-term treatment modalities are associated with lasting effects on RMR or physical activity.

Collectively, the available literature supports the notion that energy balance parameters may be altered in BCS. There is modest evidence that self-reported dietary intake improves (e.g., decreased EI, higher intake of fruits/vegetables) after active treatment for breast cancer; however, the reliance on self-reported dietary intake and small sample sizes negatively impacts the significance of these findings. Limited evidence suggests that BCS have higher RMR than matched controls, but this may be negated by decreased physical activity. There are several mechanisms that may underpin changes in energy balance among BCS, including estrogen suppression, body composition (and the resultant interaction between energy intake and expenditure), and psychological alterations, as discussed below.

## 4. Discussion

### 4.1. Estrogen Suppression in the Regulation of Energy Balance

**Figure 1:**
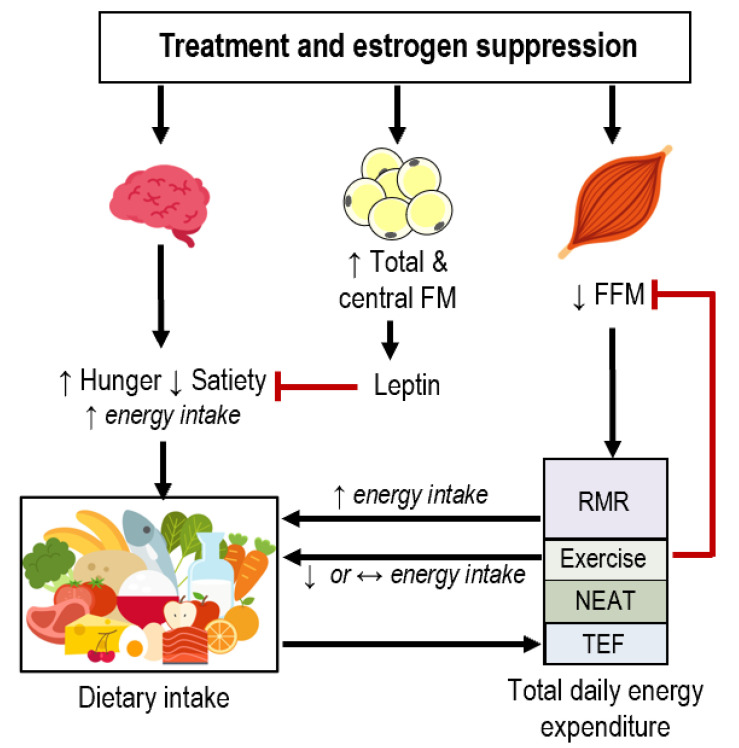
Theoretical interaction of dietary intake and components of energy expenditure after treatment and during estrogen suppression therapy. Estrogen suppression initiates changes in appetitive hormones (e.g., ghrelin, cholecystokinin, peptide-YY) that interact with hypothalamic nuclei and neuronal circuits; this, in turn may alter subjective appetite in a manner favoring increased energy intake. Changes in specific components of energy balance likely underpin the propensity for fat mass (FM) gain and fat-free mass (FFM) loss in breast cancer survivors. Experimental estrogen suppression results in increased total and central FM. This in turn will increase leptin which may alter appetite in a manner favoring decreased energy intake. Experimental estrogen suppression also decreases FFM (represented as skeletal muscle, although FFM also consists of organs and non-adipose tissues); exercise helps prevent decreased skeletal muscle and assumed FFM. There is also evidence that resting metabolic rate (RMR) positively correlates to hunger and energy intake. Exercise often results in reduced or maintained energy intake in people with obesity through alterations in appetitive hormones and subjective perceptions of appetite. Dietary intake determines the thermic effect of feeding (TEF). NEAT: non-exercise activity thermogenesis.

Approximately 75% of premenopausal women with breast cancer have estrogen- and/or progesterone-receptor positive tumors (ER+, PR+) and undergo 5–10 years of estrogen suppression via gonadotropin-releasing hormone (GnRH) agonists (i.e., leuprolide or goserelin), selective estrogen receptor modulators (i.e., tamoxifen), or aromatase inhibitor therapy [56]. These therapy regimens are highly effective for reducing the risk of cancer recurrence but may also contribute to increased FM [57], especially in younger, premenopausal women. Interestingly, weight gain occurs more often in female BCS compared to male BCS [58,59], suggesting that the more pronounced reduction in estrogen that occurs in female BCS may be detrimental for weight management. To our knowledge, there are no human data on how estrogen suppression impacts components of energy balance in BCS or whether energy balance alterations occur independently of previous chemotherapy or radiation. Our understanding of the impact of sex hormones and energy balance is derived from data in women without previous breast cancer which show that estrogen impacts dietary intake through the modulation of appetite and TDEE through modulation of physical activity and RMR, Figure 1. Although other sex hormones (e.g., progesterone, testosterone) may impact specific energy balance parameters this review will focus on estrogen for brevity and relevancy, given the impact of estrogen on body composition regulation [60]; the reader is referred to previous reviews in this area for more mechanistic perspectives [61,62,63].

#### 4.1.1. Estrogen and Appetite

While the physiological mechanisms causing altered dietary intake have not been described in BCS, experimental animal models and observational human studies of estrogen suppression indicate that estrogen is an important regulator of appetite. Animal models have shown that estrogen regulates dietary intake through peripheral appetite signals by decreasing orexigenic (e.g., neuropeptide-Y, ghrelin, and melanin-concentrating hormone) and increasing anorectic (e.g., leptin, cholecystokinin) peptides [62]. These peptides interact with the hypothalamus—and in particular the arcuate nucleus—to coordinate energy balance. Estradiol (the most potent and prevalent form of circulating estrogen)stimulates anorexigenic pro-opiomelanocortin and cocaine-amphetamine-regulated transcript neuronal populations and inhibits orexigenic neuropeptide-Y and Agouti-related peptide neurons [64,65]. These mechanisms are apparent in animal models, wherein ovariectomy results in marked increases in food intake and body weight, which are reversed with exogenous estrogen [66,67].

In humans, dietary intake varies according to menstrual cycle. Specifically, EI is lowest during the periovulatory phase of the menstrual cycle when estradiol levels are high, and greatest during the premenstrual period when progesterone levels are high [61,68]. During menopause, the production of female sex hormones drops dramatically. Hunger and prospective food consumption increases during the menopausal transition and remains elevated in the early postmenopausal years [69]. However, these changes in appetite may not cause alterations in dietary intake. In a sample of 106 healthy women, EI, protein, carbohydrate, and fiber were higher in the 3–4 years before the onset of menopause [70]. However, these changes in dietary intake were self-reported, which may introduce error. Given our understanding of the role of female sex hormones in the regulation of dietary intake during the menstrual cycle and menopausal transition, it is likely that gonadal function loss due to treatment, estrogen suppression therapy, or both contribute to the development of obesity in BCS. However, the mechanisms underpinning this phenomenon have not been systematically investigated in BCS.

#### 4.1.2. Estrogen and Total Daily Energy Expenditure

Estrogen also modulates physical activity, RMR and TDEE, as supported by both animal and experimental human studies. For example, ovariectomized rats exhibit drastic reductions in TDEE as a result of diminished physical activity and RMR [71], which are reversed by exogenous estradiol administration.

There are also human data wherein premenopausal females undergo experimental ovarian hormone suppression. Short-term (6 day) GnRH antagonist administration resulted in reduced RMR (mean ± standard error: 1334 ± 36 kcal/day) compared to RMR measured in the mid-luteal phase (1405 ± 42 kcal/day) and early follicular phase (1376 ± 43 kcal/day) [72]. Longer studies of experimental estrogen suppression utilized a GnRH agonist which supresses anterior pituitary gonadotropins and gonadal sex hormones via a negative feedback loop. Using this model, 70 premenopausal women were randomized to 20 weeks of either GnRH agonist + estradiol addback or GnRH agonist + placebo (*N* = 35 each group) [73,74]. Estrogen suppression resulted in increased visceral FM and decreased FFM, which was prevented with estradiol addback [73]. There was also a decrease in RMR in the placebo group (~−50 kcal/day) that was not observed in the group that received estradiol addback. Furthermore, 24 h EE measured via whole room indirect calorimetry was also reduced by estrogen suppression (~100–110 kcal/day), but was not prevented by estradiol addback [74]. A similar follow-up study was conducted in which premenopausal women were randomized to 24 weeks of GnRH agonist (*N* = 14), GnRH agonist + aerobic exercise (*N* = 11), or placebo (*N* = 9) [75]. Although free-living TDEE (as measured by doubly labeled water) decreased 93 kcal/day and RMR decreased 59 kcal/day in the GnRH agonist group, these differences were nonsignificant within or between groups. There were also no significant alterations in physical activity energy expenditure, although these results may be due to large variability in energy expenditure components observed in this study [75]. In sum, it appears that reduced estrogen may decrease RMR and TDEE in confined settings; however, these changes may not translate to free-living settings as evidenced by results from doubly labeled water. Because data from experimental estrogen suppression are conflicting and are an imperfect memetic of sex hormone alterations due to cancer treatment and therapy, trials of energy expenditure in BCS are greatly needed to understand energy balance.

### 4.2. Relationships between Dietary Intake and Energy Expenditure in Breast Cancer Survivors

Emerging evidence in people without previous cancer have provided consistent evidence to support the notion that body composition, RMR, and physical activity predict EI and several parameters of appetite [76,77]. Adipose tissue (the largest component of FM) relates to appetite through the release of leptin. Leptin serves as a feedback mechanism that acts through hypothalamic neuropeptide and neurons to inhibit dietary intake [78]. ‘Leptin resistance’ (a decrease in sensitivity to circulating leptin) often occurs in people with obesity and would negate the relationship between leptin and appetite. More recent investigations show modest but consistent evidence that FFM also relates to appetite and dietary intake, likely as a result of the energetic demand from metabolically active tissues that make up FFM. Specifically, the FFM-EI relationship is mediated by RMR, which positively relates to meal size and EI [79]. The correlation between RMR and EI occurs independently of FM and BMI [80], although it may be less apparent in people with obesity [81]. It is believed that EI is also driven by habitual TDEE; that is, individuals with increased physical activity and RMR would be expected to have a higher EI to compensate for their higher energy requirements. For example, free-living physical activity as measured by heart rate monitors was directly and positively related to EI as measured by 7-day weighted food records in healthy adults (BMI range: 16.7–49.3 kg/m^2^) [82]. There was also an indirect positive association between physical activity and RMR, mediated by FFM [82].

Whether these relationships exist in populations that are susceptible to aberrant appetite, body composition, and/or energy expenditure—such as BCS—has not been studied. As previously discussed in this review and others [83], BCS often experience reduced FFM, which may relate to reduced RMR. In this model, it would be expected that a reduced RMR would lead to a lower drive to eat. However, there is little research investigating how dynamic changes in body composition, RMR, and physical activity affect EI in BCS. Among males in conditions of extreme negative energy balance, both FM and FFM independently and inversely associated with EI during refeeding after severe caloric restriction. The hyperphagic response after weight loss ceased only when participants had recovered 100% of their pre-weight loss FFM, at which point FM values exceeded baseline values by 74% [84,85]. In more moderate negative energy balance over a 26-week weight loss diet, there were positive associations between the proportion of FFM lost and changes in hunger and desire to eat and negative associations between change in fullness in men, but not women [86]. These sex differences may be due in part to lower levels of FFM (expressed as a percent of total body weight) in women at baseline. While the data are limited, it is conceivable that altered body composition and RMR may relate to changes in appetite and dietary intake in BCS (Figure 1), although the existence and potential magnitude of these relationships in cancer survivors are currently theoretical.

As previously discussed in this review, BCS may decrease physical activity after treatment. Low physical activity likely contributes to FM gain directly through decreased TDEE and indirectly through downstream effects on appetite and EI. Energy balance and negative energy balance are more attainable at higher levels of physical activity (i.e., “high energy flux”). In other words, increased physical activity in sedentary adults would presumably increase TDEE, resulting in a greater buffer for high EI that is inevitable in pervasive obesogenic environments. For a comprehensive review of “energy flux”, the reader is referred to Melby et al. [87]. Higher physical activity may also relate to dietary intake via the effects of exercise on appetite. Exercise interventions decrease hunger, increase satiety, reduce neuronal responses to food, and alter appetite hormones in a manner that would support lower EI [88,89,90,91,92,93]. These concepts lend credence to the notion that low physical activity in BCS may contribute to dysregulation of energy balance through low energy flux and appetite perceptions that enhance EI.

Dietary intake and TDEE are also inherently related through TEF. The magnitude of TEF is proportionate to the energy and macronutrient content of dietary intake, with protein and alcohol eliciting a greater energetic response than fat or carbohydrate [94]. Weight loss, weight gain, obesity, insulin resistance, advanced age, physical fitness, and genetic factors also contribute to TEF variability between individuals [95]. As described above, many BCS report decreased EI after treatment and diagnosis with or without changes in macronutrient distributions, which would impact TEF. However, measuring TEF is burdensome; as a result, there are limited data on TEF in cancer patients. To date, only one study has measured TEF in breast cancer patients (*N* = 18) actively undergoing chemotherapy. TEF was defined as the increase in energy expenditure above RMR after consumption of a nutritional supplement (5 mL/kg body weight) [32]. TEF trended towards decreasing during chemotherapy and rebounded to pre-treatment levels after chemotherapy [32]. While TEF might be lower than expected during treatment, the specific interactions between nutrient digestion, absorption and metabolism and the impact on the TEF in BCS after treatment has not been explored.

### 4.3. Psychological Alterations and Energy Balance after Breast Cancer

Breast cancer diagnosis and treatment may serve as a “teachable moment” and catalyst for altering energy balance through positive health behavior changes [96]. Concerns of cancer recurrence or mortality are common among BCS, and many report feelings of fear, depression and anxiety towards their cancer prognosis, body image concerns, sexual dysfunction, work and family life problems during the transition from active treatment to long-term survivorship [97]. These psychological alterations may serve as the impetus for behavior change in sub-groups of survivors. Specifically, BCS who believe that unhealthy dietary intake, lack of physical activity, and smoking contributed to their cancer or are related to recurrence are more apt to positively modify behavior [98]. In a sample of 250 women with non-metastatic breast cancer, those who made positive changes in their dietary intake in the year after diagnosis were more likely to be younger, have lymph node involvement, be receiving adjuvant therapy, and to be more distressed at diagnosis [99]. The latter finding suggests that those with greater amounts worry about their disease and recurrence are more likely to make lifestyle changes. Qualitative data in breast, prostate, and colon cancer survivors support this notion; beliefs that behavior influences recurrence are associated with implementing positive health changes [100]. However, other data in BCS have not reported changes in other health behaviors such as tobacco or alcohol use [101], casting doubt on the applicability of the “teachable moment” for other health behavior changes. It is possible that cancer diagnosis and treatment may indeed serve as a motivator for altering dietary intake and physical activity in certain groups of BCS; however, whether these behavior changes are indelible or explain the findings presented in this review is not clear.

### 4.4. Areas for Future Research and Conclusions

There are compelling and numerous data that describe body composition alterations in BCS and there is growing consensus that diet and/or exercise interventions can prevent unfavorable changes in body composition. However, the behavioral and physiological mechanisms of energy balance in BCS are largely uncharacterized. There are several knowledge gaps that future research should address, such as:

- Expanded use of more accurate techniques such as doubly labeled water (^2^H_2_ and ^18^O), accelerometers and whole-room indirect calorimetry would help promote further understanding of TDEE and its components in different clinical populations. While these techniques are not practical in large sample sizes, they could provide useful insight on the mechanistic underpinnings of energy balance in BCS (and cancer survivors in general) in smaller samples. Other techniques that include repeated measures of body composition and energy expenditure [29,30] or mathematical models [102] may also help quantify energy balance in this population.
- Use of stable isotopes to measure intake of food groups could be used to complement recall or record-based methods of dietary intake. For example, ^13^C/^12^C can be used describe intake of C_4_ plants (e.g., corn, cane sugars) and C_3_ plants (e.g., fruits and vegetables, wheat, nuts, seeds); similarly, ^15^N/^14^N can be used to characterize fish and meat intake [103,104]. Use of isotopes paired with repeated measures of dietary recall and TDEE would provide valuable insight of energy balance in BCS.
- Inter-individual variability in body composition responses to exercise suggests that individuals compensate more or less to the same intervention. In other words, some individuals may increase EI, decrease physical activity, or both in response to exercise training. Elucidating the predictors of response and whether such predictors differ in BCS will help facilitate the design of more efficacious, personalized interventions for weight management.
- Weight loss can be achieved through alterations in physical activity and dietary intake, but most individuals regain the weight they lost [105]. Physiological and psychological changes in appetite and energy expenditure in the context of an obesogenic environment underpin weight regain [106,107]. Characterization of energy balance during weight loss and maintenance in BCS—and whether this differs from individuals without previous cancer—would help generate more durable strategies for body weight management.
- Eating behavior and appetite parameters are important determinants of dietary intake. As discussed in this review, there is modest evidence that appetite fluctuates across the menstrual cycle and menopausal transition due to altered sex hormones. Elucidation of the effects of sex hormones on appetite in estrogen-suppressed BCS may support the development of more targeted nutrition interventions.
- There is increasing cross-sectional evidence that components of dietary intake and TDEE are related. Whether specific components of TDEE predict dietary intake and appetite in instances of energy imbalance is unclear in the general population and in people with chronic disease. Elucidating the complex interrelations among energy balance parameters in the context of different conditions may help better predict intervention response and devise better solutions for weight management.
- Differentiation of outcomes according to tumor pathology (i.e., ER, PR, and human epidermal growth factor-2 status), patient age, and treatment modalities may also promote personalized intervention strategies. As previously reviewed [57,108], women who are premenopausal at diagnosis have a higher risk of FM gain compared to women who were postmenopausal at diagnosis. This is likely a direct result different treatment modalities and estrogen status; how these factors impact behavior and physiology related to energy balance is unknown.Finally, characterizing energy balance components in other cancer populations is warranted, especially in those that often undergo rigorous chemotherapy or hormonal treatments or are at risk for developing obesity (e.g., colorectal, prostate, ovarian cancers). This review focused on BCS because of the risk of weight gain, effect of hormonal therapies, and the availability of enough evidence to form conservative conclusions regarding dietary intake and energy expenditure. However, there is limited data on how various cancer types and treatment modalities may impact specific components of energy balance after treatment in other cancer types; it is also unclear if energy balance differs among cancer types or compared to individuals without previous cancer.

## 5. Conclusions

In conclusion, this review highlighted the paucity of data investigating longitudinal and comparative studies of dietary intake and energy expenditure among BCS. The majority of BCS will undergo estrogen suppression, which likely impacts dietary intake, RMR, and physical activity. Because obesity is associated with prevention and survivorship of numerous cancer types, it is imperative to elucidate factors contributing to the regulation of energy balance in cancer survivors—especially in those with increased risk of developing obesity (e.g., breast, prostate, colorectal). A more comprehensive framework of energy balance in cancer survivors will support the development of evidence-based and personalized behavioral weight management programs.

## Figures and Tables

**Table 1:** Search strategy for identification of relevant articles in a critical review.

Term Group	“Breast Cancer”	AND	“Diet”	OR	“Energy Expenditure”	OR	“Physical Activity”
**Specific search terms**	“breast cancer” OR “breast carcinoma” OR “breast neoplasm” OR “mammary”		Diet * OR nutrition * OR food * OR eating * OR appetite * OR feeding		“energy expenditure” OR “metabolic rate” OR calorimet *		“physical activity” OR exercise OR “activity monitor *” OR acceleromet * OR “activity tracker”

Terms were limited to titles and abstracts in PubMed and Web of Science. Only peer-reviewed studies (no conference abstracts) published in English were included.

**Table 2:** Dietary intake in breast cancer survivors in longitudinal or comparative studies.

Reference	Population	Dietary Intake Methods	Time Points	Main Results	
				Food-Based Results	Macronutrient-Based Results
Lei et al., 2018 [16] *	*N* = 1112 Chinese BCS with previous stage 0-III cancer; 52.2% pre- or peri-menopausal; 73.6% ER+, 57.1% PR+; 20% had overweight **, 26.7% had obesity ** at baseline	12-month FFQ; interviewer administered with photographs, portion sizes Food items were combined into 19 groups. Average daily intake of energy and macronutrients were calculated using the Chinese Food Composition Table Both expressed as median [IQR] of food serving or nutrient/1000 kcal/day	Baseline (0–12 months after diagnosis), 18- and 36 months after diagnosis *	**Increased:** Whole grains, refined grains, eggs, fruits, vegetables, potatoes, nuts **Decreased:** Cakes, poultry, red meat, processed meat, dairy, soy foods, salted foods, oil and fat, tea	*Presented as median [interquartile range]/1000 kcal/day, baseline and 36 months after diagnosis:* **Increased:** Carbohydrates, g: 121 (31); 136 (28), *p* < 0.001 Fiber, g: 8.4 (2.6); 11.0 (3.4), *p* < 0.001 **Decreased:** EI, kcal/day: 1617 (718); 1307 (537), *p* < 0.001 Protein, g: 46 (10); 42 (9), *p* < 0.001 Total fat, g: 39 (11); 35 (11), *p* < 0.001 Cholesterol, g: 173 (89); 152 (80), *p* < 0.001
Lohmann et al., 2017 [17]	*N* = 285 BCS treated in Toronto with early stage cancer (T1-3, N0-1, M0; stages not reported); 62.1% pre- or peri-menopausal; 70.5% ER/PR+; baseline BMI: 24.1 (IQR: 21.6–45.1) kg/m^2^; follow-up BMI: 25.6 (IQR:22.9–29.2) kg/m^2^ (*p* < 0.0001 for change) *N* = 167 age-matched women without previous cancer; BMI not reported	12-month Block FFQ No details on food or nutrient extraction from FFQ	Median 12.3 (range 9.4–17.6) years after diagnosis	**No change:** Fruit and vegetable servings/day (0.18 [−1.51, 2.06], *p* = 0.30)	*Presented as median [interquartile range] change* **Increased:** Carbohydrates, % EI: 3.2 (−1.5, 10.3), *p* < 0.001 Protein, % EI: 1.0 (−1.1, 3.0), *p* < 0.001 Alcohol, % EI: 0.1 (−0.5, 1.4), *p* = 0.006 Saturated fat, g/day: −7 (−15, −2), *p* < 0.001 **Decreased:** EI, kcal/day: −283 (−604, 98), *p* < 0.001 Fat, % EI: −2.6 (−8.8, 2.5), *p* < 0.001 Fat, g/day: −14 (−35, 4), *p* < 0.001 Carbohydrates, g/day: −18 (−60, 31), *p* = 0.003 Total fiber, g/day: 3.8 (−0.4, 8.8), *p* < 0.001
Shaharudin et al., 2013 [18]	*N* = 116 BCS in Malaysia; baseline BMI: 26.8 ± 5.3 kg/m^2^; follow-up BMI: 26.4 ± 5.3 kg/m^2^ (*p* = 0.029 for change); baseline body weight: 63.2 ± 13.1 kg; follow-up body weight: 62.2 ± 13.0 (*p* = 0.022)	Semiquantitative FFQ validated in Malaysians with portion sizes Food composition obtained manually	2 years after diagnosis	*N*/A	*Presented as mean ± SD pre-diagnosis and 2 years after diagnosis:* **Increased:** Carbohydrate, % EI: 57.5 ± 4.0; 61.6 ± 4.6, *p* < 0.001 **Decreased:** EI, kcal/day: 1784 ± 266; 1620 ± 380, *p* < 0.001 Protein, g/day: 67 ± 12; 59 ± 12, *p* < 0.001 Fat, g: 55 ± 12; 43 ± 15, *p* < 0.001 Fat, % EI: 27.4 ± 3.4; 23.5 ± 4.7, *p* < 0.001 Saturated fat, g: 25 ± 5; 20 ± 7, *p* < 0.001 **No change:** Protein, % EI: 15.1 ± 1.9; 14.8 ± 2.3 (exact *p* not reported) Carbohydrate, g/day: 256 ± 39; 249 ± 61 (exact *p* not reported)
Shi et al., 2020 [19]	*N* = 2865 women with invasive breast cancer; 26% premenopausal, 99% stage I-III, 84% ER/PR+; 30% had overweight, 30% had obesity	139-item modified version of the Block FFQ No details on food or nutrient extraction from FFQ Food analysis of fruits and vegetables, dietary fat, and alcohol converted to grams of ethanol Used group-based trajectory modeling to create participant groups according to nutrient intake at baseline (low, medium, high) and direction of change (increase, decrease, maintainer)	Diagnosis, 6- and 24 months after diagnosis	**Increased:** *Fruit and vegetable intake:* *N* = 320 (11%) “high baseline—increase”; 0.4–0.5 servings/day increase at 6 and 24 months *N* = 1180 (41%) “medium baseline—increase”; 0.2–0.3 servings/day increase at 6 and 24 months *N* = 1365 (48%) “low baseline—increase”; 0.2–0.3 servings/day increase at 6 and 24 months **Decreased:** *Alcohol:* *N* = 137 (5%) “high baseline—temporary decrease”; −6 g ethanol/day decrease at 6 months only *N* = 459 (16%) “medium baseline—temporary decrease”; −5 g ethanol/day decrease at 6 months only **No change:** *Alcohol:* *N* = 2269 (79%) “low baseline—maintainer”	**No change:** *Fat intake:* *N* = 131 (5%) “high baseline—maintainer” *N* = 1142 (40%) “medium-high baseline—maintainer” *N* = 1336 (47%) “medium-low baseline—maintainer” *N* = 256 (9%) “low baseline—maintainer
Velentzis et al., 2020 [20]	*N* = 1560 women with invasive stage I-III breast cancer in the United Kingdom; 32.3% pre/perimenopausal; 33.3% had overweight, 21.1% had obesity	Two 145-item semi-quantitative FFQs at study visit: one for (recalled) dietary intake in the year before diagnosis and one for dietary intake since diagnosis. Food items combined into standard food groups. Average daily intake of energy and macronutrients were calculated using the Compositional Analyses from Frequency Estimations Software	Recall of dietary intake before diagnosis; follow-up 9–15 months after diagnosis	**Increased:** Fruits, vegetables, fruit/vegetable juices, legumes, poultry, soy meat, white fish, shellfish, whole grains, cold breakfast cereal, potatoes, milk, nuts, tea **Decreased: ** Red meat, processed meat, refined grains, chips, pizza, full fat dairy, butter, desserts, chocolate, coffee, wine, other alcohol, high energy drinks	**Increased:** Fiber, g/1000 kcal EI/day: 9.6 ± 3.3; 10.1 ± 3.7 **Decreased: ** EI, kcal/day: 1893 ± 625; 1720 ± 559, *p* < 0.0001 *Presented as g/1000 kcal EI/day:* Fat: 37 ± 7; 33 ± 11, *p* < 0.0001 Saturated fat: 14 ± 4; 12 ± 5, *p* < 0.0001 Protein: 44 ± 8; 42 ± 11, *p* < 0.0001 Carbohydrate: 122 ± 19; 117 ± 32, *p* < 0.0001
Wayne et al., 2020 [21]	*N* = 260 women with newly diagnosed stage 0-IIIA breast cancer in the United States; 32.2% had overweight, 23.3% had obesity; baseline body weight: 69.3 ± 13.7 kg, follow-up body weight: 70.8 ± 14.1 kg, *p* < 0.001	114-item FFQs; baseline FFQs were for (recalled) dietary intake in the year before diagnosis No details on food or nutrient extraction from FFQ	Within 9 months of diagnosis; 2-year follow-up	*Reported as change from baseline to 2-year follow-up* **No change:** Fruit, servings/day: 0.0 ± 0.9, *p* = 0.531 Vegetables, servings/day: 0.1 ± 1.0, *p* = 0.506	*Reported as change from baseline to 2-year follow-up:* **Increased:** Protein, % EI: 0.6 ± 3.4, *p* = 0.001 Fat, % EI: 1.0 ± 6.3, *p* = 0.010 **Decreased:** EI, kcal/day: −137 ± 441, *p* < 0.001 Protein, g/day: −3 ± 23, *p* = 0.010 Carbohydrate, g/day: −21 ± 53, *p* < 0.001 Carbohydrate, % EI: −1.1 ± 8.7, *p* = 0.026

* Results from baseline to 18 months were similar to baseline-36 month comparison; for brevity, only 36 month macronutrients results are reported. ** overweight defined as BMI 23–24.9 kg/m^2^ and obesity defined as BMI ≥ 25 kg/m^2^. Otherwise, BMI 25–29.9 kg/m^2^ was classified as overweight and BMI > 30.0 kg/m^2^ was classified as obesity. BCS: breast cancer survivors; BMI: body mass index; EI: energy intake; ER: estrogen receptor; FFQ: food frequency questionnaire.

**Table 3:** Physical activity and sedentary parameters as measured by accelerometers in breast cancer survivors.

Reference	Population	Physical Activity Methods	Time Points	Main Results
Broderick et al., 2014 [40]	*N* = 24 BCS who had completed >80% of chemotherapy for stage *N* = 20 age- and education-matched women	RT3 accelerometer; worn on the waist for 7 days for sedentary, light, MVPA expressed in hours/day	6 weeks, 6 months, and 1 year after adjuvant chemotherapy completion	Non-significant trends in ↑ sedentary behavior and ↓ light activity and MVPA Control group had greater time in light activity than BCS at 6 weeks (control: 6.5 ± 1.2 vs. BCS: 5.1 ± 1.5 h/day) and 12 months (BCS: 5.0 ± 1.5 h/day), *p* = 0.003
Phillips et al., 2015 [41]	*N* = 398 BCS, stage I-IV, 14.1% premenopausal *N* = 1120 non-cancer controls block-matched for ethnicity, age, and education	Actigraph accelerometer (model GT1 M in BCS; model 7164 in controls); worn on the hip for 7 days for sedentary, total PA and time spent in light PA, ‘lifestyle’ PA, and MVPA expressed in min/day and % total time	*N*/A—cross sectional	*Presented as mean ± standard error* BCS had lower total PA (283 ± 4 vs. 347 ± 6 min/day), light PA (199 ± 2 vs. 259 ± 4 min/day), lifestyle PA (62 ± 2 vs. 72 ± 3 min/day) and MVPA (22 ± 1 vs. 16 ± 1 min/day) compared to controls (all *p* < 0.001). BCS spent higher % of time as sedentary (66.4 vs. 59.1%. *p* < 0.001) and MVPA (2.6 vs. 1.8%, *p* < 0.001) and lower % time in light PA (23.7 vs. 30.9%, *p* < 0.001) and lifestyle PA (7.4 v. 8.4%, *p* = 0.002) compared to controls.
Sabiston et al., 2014 [42]	*N* = 177 BCS, 0–20 weeks after completing primary treatment for stage I-III disease; 18.1% premenopausal	Actigraph GT3 X accelerometer; worn on the hip 7 days for sedentary and MVPA expressed in absolute min/day and % time Also expressed as percentage of participants meeting MVPA guidelines: ≥150 min MVPA/week or ≥75 min vigorous activity/week	Baseline (3.49 ± 2.36 months since treatment completion) and 3-,6-, 9-, and 12 months after baseline	No change in sedentary time MVPA decreased over time (16.3 ± 12.1 min/day at baseline; 14.2 ± 11.4 min/day at 12-month follow-up, *p* = 0.01). 29% of survivors met MVPA guidelines at baseline and 22% met guidelines at 12 months. BCS with higher waist-to-height ratio and higher BMI engaged in less MVPA.
Shi et al., 2017 [43]	*N* = 241 BCS who had completed chemotherapy or radiotherapy, 1–3 years after diagnosis *N* = 741 healthy adults > 35 years	Actigraph GT3 X accelerometer; worn on the hip for 7 days for sedentary, light, MVPA, and number of sedentary bouts >20 min, expressed in min/day	*N*/A—cross sectional	MVPA was higher in BCS vs. controls (29 [95% CI: 26 to 31] vs. 22 [20 to 24] min/day, *p* < 0.001) Trend towards greater sedentary bouts in the BCS vs. controls (180 [169 to 190] vs. 168 [160 to 175], *p* = 0.08)
Tabaczynski et al., 2021 [44]	*N* = 20 BCS, stage I-IIIa disease, 77.9 ± 42.7 months post-diagnosis; 85.0% white *N* = 20 age-matched healthy controls; 75% white	Actigraph GT3 X accelerometer; worn on the waist for 7 days for sedentary, light PA, and MVPA expressed in min/day. Also expressed as percentage of participants meeting MVPA guidelines: ≥150 min MVPA/week	*N*/A—cross sectional	BCS spent less time in sedentary activities (491 ± 79 vs. 588 ± 74 min/day *p* = 0.046).
Yee et al., 2014 [45]	*N* = 71 BCS with stage IV disease, 2.9 ± 3.1 years after diagnosis *N* = 71 healthy control women without previous cancer	SenseWear monitor; worn on upper arm for 7 days for steps/day and time spent in MVPA, expressed in min/day	*N*/A—cross sectional	BCS had less steps/day (5434 ± 3174 vs. 9635 ± 3327, *p* < 0.001) and MVPA (82 ± 78 vs. 142 ± 82 min/day, *p* < 0.001) compared to controls.

Values expressed as mean ± standard deviation unless otherwise noted. BCS: breast cancer survivors; CI: confidence interval; MVPA: moderate-to-vigorous physical activity; PA: physical activity

## Data Availability

Not Applicable.
